# Beneficial Effect of Quercetin on Erythrocyte Properties in Type 2 Diabetic Rats

**DOI:** 10.3390/molecules26164868

**Published:** 2021-08-11

**Authors:** Tomas Jasenovec, Dominika Radosinska, Marta Kollarova, Peter Balis, Kristina Ferenczyova, Barbora Kalocayova, Monika Bartekova, Lubomira Tothova, Jana Radosinska

**Affiliations:** 1 Faculty of Medicine, Institute of Physiology, Comenius University in Bratislava, Sasinkova 2, 813 72 Bratislava, Slovakia; tomas.jasenovec@fmed.uniba.sk (T.J.); marta.husseinova@fmed.uniba.sk (M.K.); monika.bartekova@savba.sk (M.B.); 2 Faculty of Medicine, Institute of Immunology, Comenius University in Bratislava, Odborarske Namestie 14, 811 08 Bratislava, Slovakia; dominikaradosinska@gmail.com; 3 Centre of Experimental Medicine, Slovak Academy of Sciences, Dúbravská Cesta 9, 841 04 Bratislava, Slovakia; peter.balis@savba.sk (P.B.); kristina.ferenczyova@savba.sk (K.F.); barbora.kalocayova@savba.sk (B.K.); 4 Faculty of Medicine, Institute of Molecular Biomedicine, Comenius University in Bratislava, Sasinkova 4, 811 08 Bratislava, Slovakia; lubomira.tothova@imbm.sk

**Keywords:** erythrocyte, erythrocyte deformability, nitric oxide, Zucker diabetic fatty rats, ZDF, quercetin, diabetes mellitus, age

## Abstract

Diabetes mellitus is characterized by tissue oxidative damage and impaired microcirculation, as well as worsened erythrocyte properties. Measurements of erythrocyte deformability together with determination of nitric oxide (NO) production and osmotic resistance were used for the characterization of erythrocyte functionality in lean (control) and obese Zucker diabetic fatty (ZDF) rats of two age categories. Obese ZDF rats correspond to prediabetic (younger) and diabetic (older) animals. As antioxidants were suggested to protect erythrocytes, we also investigated the potential effect of quercetin (20 mg/kg/day for 6 weeks). Erythrocyte deformability was determined by the filtration method and NO production using DAF-2DA fluorescence. For erythrocyte osmotic resistance, we used hemolytic assay. Erythrocyte deformability and NO production deteriorated during aging—both were lower in older ZDF rats than in younger ones. Three-way ANOVA indicates improved erythrocyte deformability after quercetin treatment in older obese ZDF rats only, as it was not modified or deteriorated in both (lean and obese) younger and older lean animals. NO production by erythrocytes increased post treatment in all experimental groups. Our study indicates the potential benefit of quercetin treatment on erythrocyte properties in condition of diabetes mellitus. In addition, our results suggest potential age-dependency of quercetin effects in diabetes that deserve additional research.

## 1. Introduction

Red blood cells (RBCs) are the most numerous blood elements responsible for the transport of respiratory gases in the organism. They are supposed to repeatedly pass through the capillaries with a smaller diameter than that of RBCs. To achieve this, RBCs are deformable, i.e., they are able to customize their shape by squeezing and re-assume their normal biconcave disc shape dependently of the vessel diameter and blood flow. Diabetes mellitus (DM) is a condition that is unfavorable for the quality of RBCs [1,2]. It was shown that impairment in RBC properties in individuals suffering from DM is involved in the development of endothelial dysfunction [3] and typical microvascular complications, such as diabetic foot [4], retinopathy [5,6] or nephropathy [5]. It is generally accepted that oxidative stress is involved in the development of DM and its undesirable consequences [7,8]. Quercetin (QCT) has been suggested to protect individuals from the development of DM, as well as from complications resulting from this disorder [9]. The antioxidant properties of QCT are based on its ability to directly scavenge free radicals, as well as to regulate enzyme-mediated (e.g., superoxide dismutase, catalase, glutathione peroxidase) and enzyme-independent (e.g., glutathione levels) antioxidant defense of the organism. QCT modulates multiple signaling pathways (e.g., the inhibition of inducible nitric oxide (NO) synthase and xanthine oxidase activity, as well as tumor necrosis alpha production and gene expression), leading to maintaining the oxidative balance—for a review, see [10,11]. QCT belongs to the intensively studied antioxidants; according to the Pubmed database (May 2021), there are more than 21 thousand results for the keyword “quercetin” and more than a thousand reviews related to this compound. In addition, Pubmed database showed more than a thousand papers for “quercetin and diabetes”. Nevertheless, only 15 of them were focused on RBC characteristics, and none are available regarding the “quercetin diabetes erythrocyte deformability”.

One possible way to study the impact of type 2 DM on RBC properties or to test the efficiency of QCT treatment in condition of DM is to take advantages of the Zucker diabetic fatty (ZDF) rat model. It is a well-characterized model showing typical features of the human disease—hyperphagia, obesity, hyperinsulinemia and hyperglycemia [12]. In prediabetic ZDF rats (7-week-old animals), RBCs were not able to deliver adequate oxygen supply to skeletal muscle [13]. Other RBC-related pathologies in ZDF rats include an increase in RBC aggregation and a decrease in mean cell volume [14]. Various factors are involved in the maintenance and regulation of RBC deformability. RBCs possess endothelial NO synthase and produce their own NO in such a way as to regulate deformability. QCT exhibited a protective effect on NO bioavailability in aorta under the condition of DM [15]. However, its effect on NO production by RBCs is not elucidated yet.

Oxidative stress that is to a high extent implicated in DM pathophysiology is also a significant factor influencing the quality of RBCs [16]. According to the free radical theory of aging, oxidative damage of cells and tissues has been suggested as the cause of age-related decline in body functions. With increasing age, the risk of DM development, as well as DM complications generally increases. Furthermore, aging itself is related to the worsening of RBC functions, including their deformability [1]. Importantly, it has been shown that therapy beneficial in younger individuals may not have the same effect in aged organisms [17]. Thus, it is worthy to investigate the treatment efficiency in distinct age categories.

Considering all facts mentioned above, we aimed to investigate the effect of QCT on selected RBC properties—RBC deformability, NO production by RBCs and RBC osmotic resistance—under the monitoring of parameters of oxidative stress in younger prediabetic (6-month-old) and older diabetic (1-year-old) ZDF rats.

## 2. Results

### 2.1. Basic Characteristics of Experimental Animals

**Figure 1:**
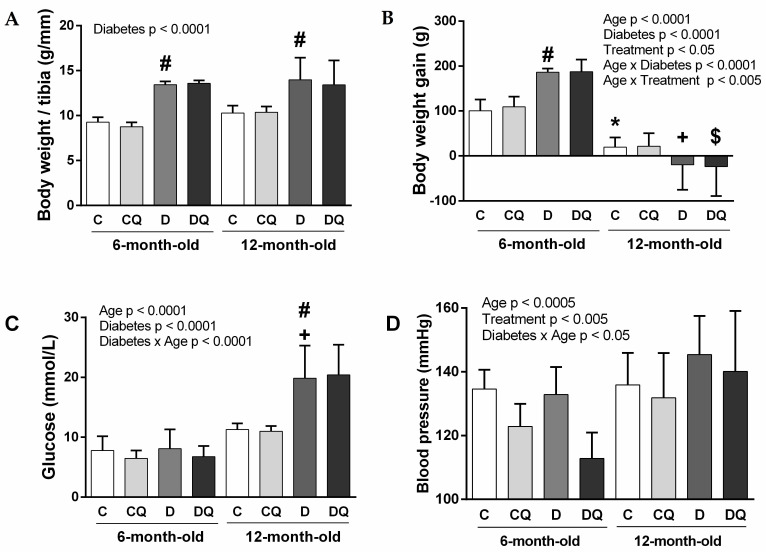
Basic characteristics of experimental animals: body weight/tibia ratio (**A**), body weight gain (**B**), blood glucose concentration (**C**), systolic blood pressure (**D**). Abbreviations: C—lean control vehicle-treated, CQ—lean control quercetin-treated, D—Zucker diabetic fatty (ZDF) vehicle-treated, DQ—ZDF quercetin-treated. * *p* < 0.05 versus 6-month-old C group, + *p* < 0.05 versus 6-month-old D group, # *p* < 0.05 versus age-matched C group, $ *p* < 0.05 versus age-matched D group. Data are presented as means ± standard deviations.

Body weight/tibia (BW/T) ratio (Figure 1A) was higher in diabetic rats compared with non-diabetic ones of both age categories. QCT treatment did not influence BW/T ratio in all experimental groups.

BW gain (Figure 1B) was lower in 12-month-old compared with 6-month-old and higher in 6-month-old diabetic compared with 6-month-old control animals. Regarding the BW gain and QCT treatment, 3-way ANOVA revealed significance for factor treatment, as well as for the interaction of age × treatment. In young animals, BW gain was higher after QCT treatment, which was not consistently observable in older animals.

Blood glucose analysis (Figure 1C) showed higher glycemia in older diabetic compared with older control rats. QCT treatment did not influence glycemia in all experimental groups.

For systolic blood pressure (Figure 1D), age and treatment factors, as well as the interaction of diabetes × age were significant. According to 3-way ANOVA, QCT treatment resulted in a decrease in blood pressure values in experimental animals.

**Figure 2:**
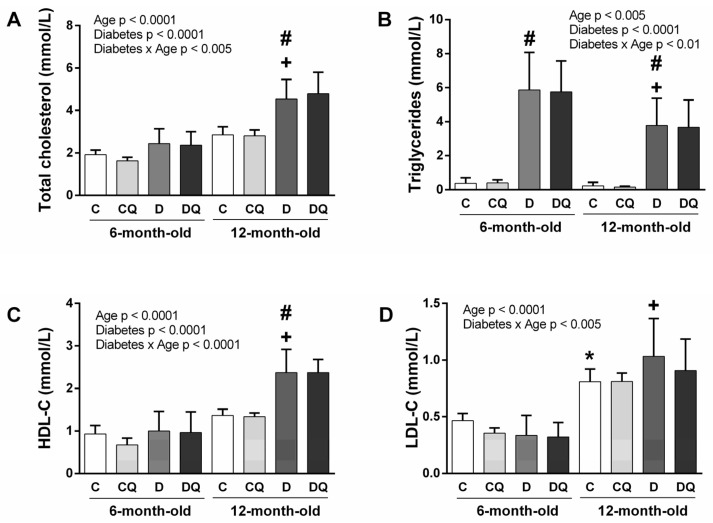
Parameters of lipid metabolism. Concentration of total cholesterol (**A**), triglycerides (**B**), HDL-cholesterol (**C**), LDL-cholesterol (**D**). Abbreviations: C—lean control vehicle-treated, CQ—lean control quercetin-treated, D—Zucker diabetic fatty (ZDF) vehicle-treated, DQ—ZDF quercetin-treated. * *p* < 0.05 versus 6-month-old C group, + *p* < 0.05 versus 6-month-old D group, # *p* < 0.05 versus age-matched C group. Data are presented as means ± standard deviations.

Regarding the parameters of lipid metabolism (Figure 2), the significance for diabetes and age factors, as well as for the interaction of diabetes × age in concentration of total cholesterol (Figure 2A), triglycerides (TAG) (Figure 2B) and HDL-cholesterol (HDL-C) (Figure 2C) was observed.

For LDL-cholesterol (LDL-C), the significance was observable only for the age factor and for the interaction of age × diabetes (Figure 2D). Statistical analysis revealed an increase in total cholesterol, HDL-C and LDL-C, but a decrease in TAG concentrations in 12-month-old animals compared with 6-month-old ones. Diabetic rats had higher TAG, total cholesterol and HDL-C when compared with controls. The administration of QCT did not influence observed parameters of lipid metabolism.

**Figure 3:**
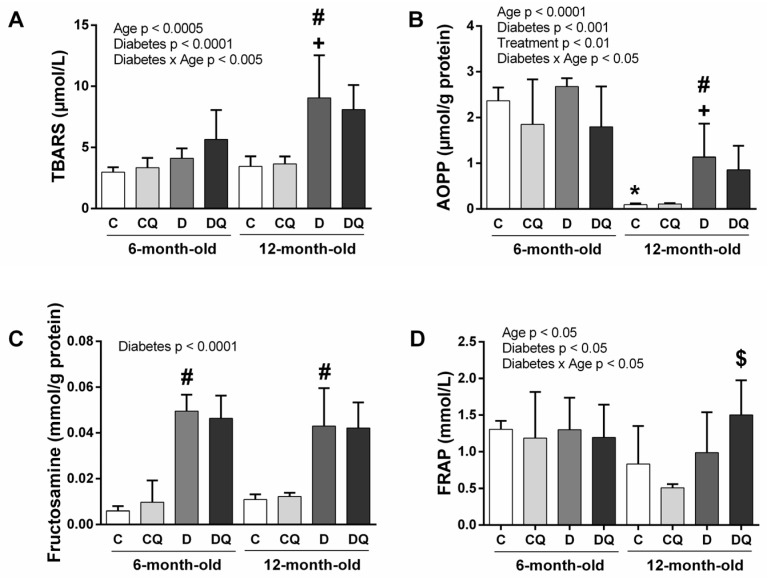
Parameters of oxidative stress and antioxidant status determined by ferric reducing antioxidant power (FRAP) in blood plasma: thiobarbituric acid reactive substances (TBARS) (**A**), advanced oxidation protein products (AOPP) (**B**), fructosamine (**C**), FRAP (**D**). Abbreviations: C—lean control vehicle-treated, CQ—lean control quercetin-treated, D—Zucker diabetic fatty (ZDF) vehicle-treated, DQ—ZDF quercetin-treated. * *p* < 0.05 versus 6-month-old C group, + *p* < 0.05 versus 6-month-old D group, # *p* < 0.05 versus age-matched C group, $ *p* < 0.05 versus age-matched D group. Data are presented as means ± standard deviations.

Focusing on the parameters of oxidative stress, analysis of markers of lipid peroxidation (thiobarbituric acid reactive substances—TBARS) revealed significance for diabetes and age factors, as well as for the interaction of diabetes × age. In 1-year-old animals, TBARS concentration was higher in diabetic versus control animals, independently of QCT treatment (Figure 3A).

Three-way ANOVA showed lower advanced oxidation protein product (AOPP) levels after QCT treatment in experimental animals without differences among groups in a multiple comparison test. In older animals, significantly higher AOPP levels were observed in the diabetic group compared with the control one (Figure 3B). A marker of carbonyl stress, fructosamine, was higher in diabetic animals of both ages without the effect of QCT treatment (Figure 3C). Regarding the plasma antioxidant capacity determined by ferric reducing antioxidant power (FRAP) (Figure 3D), despite the higher data variability, diabetes and age factors, as well as the interaction of these factors, were significant. In a multiple comparison test, FRAP was higher in 12-month-old diabetic rats treated by QCT in comparison with age-matched diabetic controls.

### 2.2. RBC Deformability

**Figure 4:**
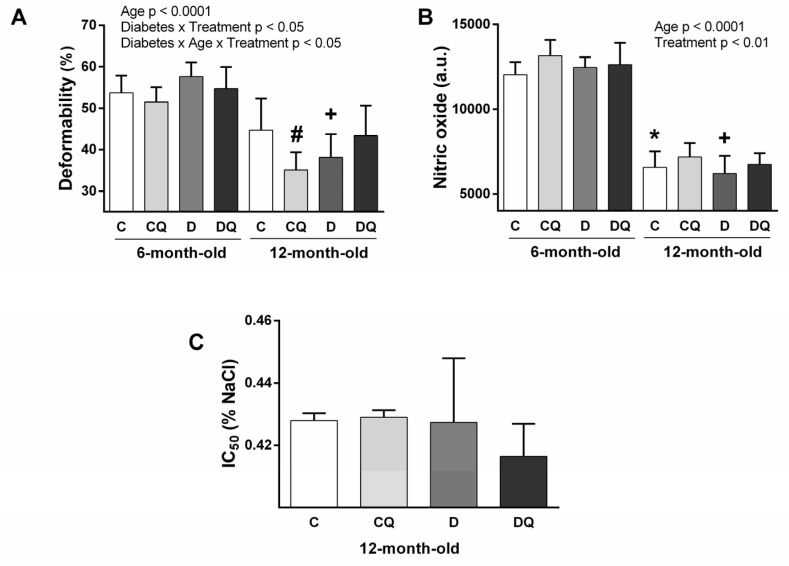
Parameters of erythrocytes: erythrocyte deformability (**A**), nitric oxide production by erythrocytes (**B**), osmotic resistance of erythrocytes determined by the concentration of NaCl providing 50% hemolysis—IC_50_ (**C**). Abbreviations: C—lean control vehicle-treated, CQ—lean control quercetin-treated, D—Zucker diabetic fatty (ZDF) vehicle-treated, DQ—ZDF quercetin-treated. * *p* < 0.05 versus 6-month-old C group, + *p* < 0.05 versus 6-month-old D group, # *p* < 0.05 versus age-matched C group. Data are presented as means ± standard deviations.

The analysis showed the significance for age and the interaction of diabetes × QCT treatment, as well as for the interaction of diabetes × QCT treatment × age. RBC deformability was lower in 12-month-old animals compared with 6-month-old ones. Three-way ANOVA suggested different response to QCT treatment in younger and older rats. In older animals, the different response of control and diabetic animals to QCT treatment was observed (Figure 4A); worsening RBC deformability was observable in control, but a tendency for improvement (*p* = 0.06) was observed in diabetic individuals.

### 2.3. NO Production in RBCs

NO production by RBCs was lower in older animals independently of diabetes and QCT treatment. Three-way ANOVA analysis suggested improvement of this RBC property after QCT treatment independently of age or diabetes (Figure 4B).

### 2.4. Osmotic Resistance of RBCs

These measurements were performed using RBCs from 12-month-old animals only. Statistical analysis revealed improvement in RBC resistance against hypotonic environment in diabetic animals (not in controls) after QCT treatment in concentration range 0.5–0.45% of NaCl (*p* = 0.02 for 0.45% NaCl and *p* = 0.03 for 0.5% NaCl). However, analysis of IC_50_ (the concentration of NaCl resulting in 50% hemolysis) did not show any difference among the experimental groups (Figure 4C).

### 2.5. Correlations between RBC Deformability and Biometrical and Biochemical Parameters

The summary of significant correlations is presented in Table 1. In all 6-month-old diabetic rats, a negative correlation between RBC deformability and BW/T ratio (r = −0.65, *p* = 0.04) was found and the same was observable in both (younger and older) C (lean control vehicle-treated) groups (r = −0.65, *p* = 0.01), as well as in both CQ (lean control QCT-treated) groups (r = −0.63, *p* = 0.01). In all 12-month-old rats, RBC deformability was inversely related to LDL-C concentration (r = −0.41, *p* = 0.008). Concerning the antioxidant status determined by FRAP, RBC deformability positively correlated with ferric-reducing ability of plasma in all 12-month-old animals (r = 0.39, *p* = 0.02).

## 3. Discussion

Protection of RBC functions is necessary not only for RBCs themselves but is beneficial for almost any tissue or organ in the human organism. The biconcave shape allows RBCs to change shape without sustaining damage. This remarkable property—RBC deformability—significantly affects the lifespan of RBCs, blood flow, and hemodynamics in general. Alterations in RBC deformability play an important role in the pathogenesis of various diseases, e.g., cardio-vascular [1]. A decrease in RBC deformability was observed in patients suffering from DM with elevated glycated hemoglobin [18] and was also associated with DM complications [4,5,6]. Hyperglycemia leads to tubulin acetylation and its translocation to the RBC membrane leads to a reduction in RBC deformability, as well as to increased osmotic fragility [19]. As DM is considered the free radical disease, antioxidants could be beneficial in the reduction of oxidative stress allowing one to restore or prevent the deterioration of RBC deformability.

QCT, an intensively studied antioxidant, already induced a protective effect on RBCs during in vitro condition of oxidative stress [20]. Specifically, the sodium benzoate-induced moderate oxidative stress in human RBCs was ameliorated in the presence of QCT at 10 μM concentration. However, the in vivo effect of QCT on RBCs in diabetic individuals is not known yet. Interestingly, RBC deformability was not modified or worsened after QCT administration in younger control and diabetic individuals, as well as in older control animals in our experiments. Regarding RBC deformability, only older—i.e., 1-year-old diabetic rats were able to profit from QCT administration. It may be in agreement with the observations of a previous in vitro study showing the increase in RBC membrane fluidity after QCT treatment (at concentrations of 10 and 100 μM) in hypercholesterolemic patients only but not in the RBCs of healthy volunteers [21].

Considering the age factor in the efficiency of QCT treatment (observable in RBC deformability and BW gain), in addition to comorbid diabetes, the different response to QCT treatment in distinct age categories can be expected. Thus, it is noteworthy to deal with the interaction of age versus diabetes, i.e., with the differences between the lean controls and ZDF rats in 6-month-old and 12-month-old animals. Despite the higher food intake in diabetic individuals compared with control ones (non-published data) in both age categories, the BW gain was higher in younger but lower in older diabetic rats than in age-matched controls. Another difference was also observed in plasma glucose level; hyperglycemia was more apparent in older diabetic rats, and the same was applicable for total cholesterol concentration, as well as HDL-C and LDL-C. Interestingly, TAG was lower in 12-month-old diabetic rats than in 6-month-old ones. Regarding the parameters of oxidative stress, lipid peroxidation estimated by TBARS measurement and the oxidation of proteins (AOPP parameter) were more intense in older diabetic animals. Considering the documented unequal condition of diabetic individuals of different ages, diverse responses to QCT treatment could be expected in our experiment.

RBC deformability decreased with aging in ZDF rats. The analogous age-related decline in this RBC property was shown in humans [1,22,23]. It seems to be the consequence of the cumulative effect of multiple intracellular and extracellular factors. Perhaps the most studied mechanisms responsible for the decrease in RBC deformability with age are based on the oxidative theory of aging [22]. In addition, it could be the consequence of changes in sialic acid content [24] or a decrease in Na,K-ATPase activity [25] in RBC membranes. The decrease in NO production by RBCs with aging was observed in the proposed experiment. Thus, the decrease in RBC deformability may be at least partially ascribed to a decrease in NO production by these blood elements. QCT administration has a beneficial effect on NO production by RBCs. Similar protective action of QCT treatment (at concentration 10 mg/kg of BW/day for 6 weeks) on NO bioavailability was described in the aorta [15]. In our study, the effect of QCT treatment on osmotic resistance characterized by IC_50_ (concentration of NaCl resulting in 50% hemolysis) was not detected. However, in older QCT-treated diabetic individuals, RBCs were more able to resist the hypotonic environment in the narrow range of NaCl concentration than RBCs of diabetic vehicle-treated rats.

It is generally accepted that DM induces multiple abnormalities in RBCs, e.g., increased aggregation, decrease in membrane fluidity and deformability—for a review, see Radosinska and Vrbjar [1]. It is noteworthy that no statistically significant difference in RBC deformability between control and age-matched diabetic animals was determined in our study. The worsening of this property has been described in humans suffering from DM [1,26], as well as in streptozotocin-induced experimental DM [27]. To the best of our knowledge, RBC deformability in ZDF (obese fa/fa) model of DM has not been investigated yet. However, control counterparts—ZDF Rat (Lean fa/+)—exhibited significantly higher RBC aggregation in comparison with other commercially available clinically healthy rat strains. In addition, despite no statistically significant differences in RBC deformability in commonly used control strains, RBCs are smaller in lean ZDF rats. It was suggested that the lack of difference does not implicate that RBC deformability in the control ZDF lean rats is unaltered compared with other commonly used control rat strains [14]. Considering this, further studies are required to elucidate whether the ZDF rat model of type 2 DM is optimal for hemorheological studies. It is also not clear whether the RBC deformability would significantly worsen with age in the diabetic animals of the ZDF model in comparison with age-matched lean counterparts. However, what was observable in our experiments was a decrease in RBC deformability with an increase in LDL-C concentration and an increase in RBC deformability, with an increase in FRAP in 12-month-old animals. In addition, normalized BW (to tibial length) was inversely proportional to RBC deformability. Abnormalities in this property of RBCs were described in obese patients in comparison with healthy individuals as well [28]. It was also shown that RBCs from obese donors were more susceptible to hemolysis under storage compared with RBCs from nonobese donors, due to the alteration of membrane lipid composition and antioxidant dysregulation [29].

The observations of this study allowed us to evaluate multiple aspects regarding the RBC parameters, ZDF model of type 2 DM, aging and QCT treatment. Taken together, some parameters were not affected by QCT treatment (BW/T ratio, glycemia, parameters of lipid metabolism, TBARS, fructosamine, FRAP); some were consistently improved in control, as well as diabetic rats (NO production by RBCs, AOPP, systolic blood pressure), but in some of them, we observed different responses in control and diabetic individuals to QCT administration (RBC deformability, RBC osmotic resistance). Focusing on the effect of QCT on RBC properties only, an improvement in NO production by RBCs in all experimental groups was observed, and, in older diabetic animals, a weak but significant increase in osmotic stability was also observed. However, according to 3-way ANOVA analysis, RBC deformability was increased post treatment in older diabetic rats only.

## 4. Materials and Methods

### 4.1. Study Design

Male obese ZDF rats as well as their age-matched lean controls of two age categories: 6-month-old and 12-month-old animals, at the beginning of the experiment were used. Rats of both ages were assigned to 4 experimental groups: lean control vehicle-treated (C), lean control QCT-treated (CQ), obese ZDF vehicle-treated (D) and obese ZDF QCT-treated (DQ) groups. Male ZDF (fa/fa) and lean littermate controls (fa/+) were supplied by the breeding facility at the Department of Toxicology and Laboratory Animal Breeding, Centre of Experimental Medicine, Slovak Academy of Sciences, Dobra Voda, Slovak Republic. A summary of the count of rats in individual groups is presented in Table 2. In older animals, a higher count of experimental animals was used due to higher variability of glycemia in diabetic ZDF rats. In addition, we expected higher mortality in this age category that was not subsequently confirmed. QCT (Sigma-Aldrich, cat. No Q4951, St. Louis, MO, USA, declared purity by producer ≥ 95%) was dissolved in ethanol and served in the biscuit at a dose of 20 mg/kg/day for 6 weeks, as previously described [17]. As a vehicle, a corresponding small amount of ethanol in the biscuit was provided in both lean and diabetic controls (C and D groups). Since QCT is poorly water-soluble, ethanol was selected as the most convenient non-water-based solvent for oral in vivo application. More precisely, QCT was dissolved in ethanol at a concentration of 20 mg/mL before application. Then, an appropriate volume of this solution (according to individual animal BW) was applied on a piece of biscuit (prepared individually for that particular animal), and after drying, the biscuit was applied to the animal. At the end of the experiment, all experimental animals were anaesthetized with thiopental (50 mg/kg, intraperitoneally) and heparinized (500 IU, subcutaneously). Blood was taken from the abdominal aorta and collected in heparin tubes (FL Medical, Torreglia, Italy). Whole blood (10 μL) was provided for the determination of NO production by RBCs, while the rest of the blood was centrifuged (1200× *g*, 10 min, 4 °C) in order to separate RBCs for measurements of their deformability and osmotic resistance. Parameters of lipid metabolism—total cholesterol, HDL-C, LDL-C and TAG—as well as parameters of oxidative stress and antioxidant status—FRAP, AOPP, fructosamine and TBARS—were determined in blood plasma. Until analyses, blood plasma was stored at −70 °C.

### 4.2. Deformability of RBCs

RBC deformability was determined using a filtration method as described previously [30]. RBCs were washed three times in saline solution. The washed RBCs were suspended in manufacturer-formulated Cellpack solution (diluent for Sysmex blood analyzer, 1:1000, *v*:*v*, Sysmex, Slovakia) and filtered by centrifugation at 175× *g* through membrane filters with 5 μm pores in diameter (Ultrafree-MC SV Centrifugal Filter; Merck Millipore Ltd., Tullagreen Carrigtwohill, Ireland). RBC deformability was calculated as a ratio of filtered RBCs to the count of RBCs determined before filtration. The intra sample variability of this method was approximately 5–10%.

### 4.3. NO Production in RBCs

As an indicator of NO presence, DAF-2 DA was used similarly as in our previous study [30]. Whole blood was diluted 1:9 (*v*:*v*) in modified physiological salt solution (in mmol/L: NaCl 118.99, KCl 4.69, NaHCO_3_ 25, MgSO_4_.7H_2_O 1.17, KH_2_PO_4_ 1.18, CaCl_2_.2H_2_O 2.5, Na_2_EDTA 0.03, glucose 5.5, pH 7.4, all reagents provided by Sigma-Aldrich, St. Louis, MO, USA) and treated with DAF-2 DA (25 μmol/L, Abcam, Cambridge, UK) at room temperature for 10 min in the dark. Blood samples were analyzed under a fluorescence microscope (Axio Imager M2, Zeiss, Jena, Germany) using filters for fluorescein isothiocyanate (λ_ex_ = 465–495 nm, λ_em_ = 515–555 nm). The fluorescence was quantified using ImageJ 1.53e software (National Institutes of Health, Bethesda, MD, USA). More precisely, RBCs were separated using a threshold adjuster in computer-recorded images. Afterwards, all of them were filtered according to their size and circularity. RBCs passing or touching through the image edges were excluded automatically. Overlapping RBCs were removed from the analysis manually. We determined fluorescence of an average of 860 RBCs in each sample. The intensity of fluorescence is presented as integrated density corresponded to a single RBC.

### 4.4. Determination of RBC Osmotic Resistance

The RBC osmotic resistance was performed using blood samples from 1-year-old animals only (as this method requires a significant volume of blood that was not possible to obtain in younger animals). A set of 7 solutions of NaCl (Sigma-Aldrich, St. Louis, MO, USA) in the concentration range from 0.1 to 0.9% (0.1, 0.3, 0.35, 0.4, 0.45, 0.5, 0.9% of NaCl) was prepared. At the beginning, washed RBCs were suspended in saline solution (1:2, *v*:*v*). Then, 20 μL of RBC suspension were mixed with 2 mL of each NaCl solution and incubated for 30 min at room temperature. Afterwards, all samples were centrifuged (1200× *g*, 5 min, Hettich MIKRO 120 centrifuge). The degree of hemolysis was determined spectrophotometrically by measuring the absorbance of each supernatant at a wavelength of 540 nm. The absorbance of blood samples mixed with 0.1% of NaCl was used as equivalent to 100% hemolysis. IC_50_, the concentration of NaCl resulting in 50% hemolysis, was calculated.

### 4.5. Biochemical Analysis in Blood Plasma

The concentrations of glucose, total cholesterol, LDL-C, HDL-C and TAG in blood plasma were determined using BioLis 24i/CLC480 Chemistry Analyzer (Carolina Liquid Chemistries, Greensboro, NC, USA).

Biochemical analysis of oxidative status was described in more details previously in Radosinska et al. (2019) [30]. Markers of protein oxidation were measured by spectrophotometric analysis of AOPP. Markers of lipid peroxidation (TBARS) were measured spectrofluorometrically. For fructosamine measurement, 20 μL of samples and standards (1-deoxy-morpholino-D-fructose) were added to the microtiter plate. Thereafter, nitro blue tetrazolium was added, and the reaction was shortly mixed and incubated at 37 °C for 15 min. The absorbance was measured at λ = 530 nm. FRAP was measured spectrofluorometrically as the parameter reflecting the antioxidant status of experimental animals. The protein concentration used for the standardization of AOPP and fructosamine concentration were both determined by a bicinchoninic acid kit (Sigma-Aldrich, St. Louis, MO, USA), according to the manufacturer’s instructions. All above mentioned measurements (spectrophotometry and spectrofluorometry) were performed on the multidetection microplate reader Tecan Safire II (Grödig, Austria), and all reagents were obtained from Sigma-Aldrich (St. Louis, MO, USA).

### 4.6. Statistical Analyses

The data are presented as the means ± standard deviations. Normality of the data was analyzed by the D’Agostino-Pearson omnibus test. Outliers were detected using the Grubbs’ test and removed from further analyses. Statistical significance was analyzed by 3-way ANOVA with main factors: diabetes, QCT treatment and age, followed by the Tukey’s multiple comparisons test. Pearson or Spearman correlation coefficients (depending on the normality of data distribution) were calculated to explore the relationships between variables. Differences were considered as significant at *p* < 0.05. Software GraphPad Prism 8.2.1 was used for the data analysis.

## 5. Conclusions

In conclusion, the results of our study suggest potential beneficial effect of QCT administration on the quality of RBCs in condition of type 2 DM. Nevertheless, the response to QCT treatment depended on the age of the experimental animals. Thus, for QCT implementation into use in humans, it would be important to specify the class of individuals who can profit from this antioxidant administration.

## Figures and Tables

**Table 1:** Correlations between erythrocyte deformability and biometrical and biochemical parameters.

Experimental Group	Parameter	r	*p* Value
All 6-month old diabetic rats (vehicle- and QCT-treated)	BW/T ratio	−0.65	0.04
6- and 12-month-old lean vehicle-treated rats	BW/T ratio	−0.65	0.01
6- and 12-month-old lean QCT-treated rats	BW/T ratio	−0.63	0.01
All 12-month-old rats	LDL cholesterol	−0.41	0.008
All 12-month-old rats	FRAP	0.39	0.02

Abbreviations: BW/T—body weight/tibia, FRAP—ferric reducing antioxidant power, r—correlation coefficient, QCT—quercetin.

**Table 2:** The count of experimental animals in individual groups.

Experimental Group	6-Month-Old	12-Month-Old
Lean control vehicle-treated (C)	n = 7	n = 8
Lean control quercetin-treated (CQ)	n = 6	n = 12
Obese ZDF vehicle-treated (D)	n = 6	n = 15
Obese ZDF quercetin -treated (DQ)	n = 8	n = 17

Abbreviations: ZDF—Zucker diabetic fatty rats.

## Data Availability

The data presented in this study are available in this manuscript.
